# Antenatal and Neonatal Management of Siblings With Carbonic Anhydrase VA Deficiency

**DOI:** 10.1002/jmd2.70076

**Published:** 2026-02-23

**Authors:** Sophie Manoy, Tahlee Minto, Kalliope Demetriou, Matthew Lynch, Arthavan Selvanathan, Luke Jardine, Michelle Lipke, Carolyn Bursle, Anita Inwood, Jim McGill, David Coman

**Affiliations:** ^1^ Queensland Lifespan Metabolic Medicine Service, Queensland Children's Hospital Brisbane Australia; ^2^ School of Medicine The University of Queensland Brisbane Australia; ^3^ Department of Neonatology Mater Mothers Hospital Brisbane Australia; ^4^ School of Nursing and Social Work The University of Queensland Brisbane Australia; ^5^ Department of Chemical Pathology The Royal Brisbane and Women's Hospital Brisbane Australia

**Keywords:** carbonic anhydrase VA deficiency, CAVA deficiency, expectant neonatal management, inborn error of metabolism

## Abstract

Carbonic anhydrase VA (CAVA) deficiency (OMIM 114761) is an ultra‐rare inborn error of metabolism with fewer than 20 cases described. Affected infants present in the first days of life with hyperammonaemia, lactic acidosis, ketonaemia and encephalopathy. Prenatal genetic testing can facilitate the diagnosis of subsequent affected pregnancies and permit proactive clinical management to prevent metabolic decompensation. Here we describe the clinical course of two sibling infants antenatally diagnosed with CAVA deficiency who were monitored and managed in the newborn period without decompensation. The proband, their older brother, had presented on day four of life with marked lactic acidosis, hyperammonaemia and encephalopathy requiring haemofiltration due to CAVA deficiency. His brothers were each born in a tertiary neonatal setting. They were managed with regular 3–4 hourly breastfeeds with supplementary expressed breast milk and formula top‐ups to ensure optimal nutrition. In addition, they received carglumic acid (100 mg/kg daily) for 5 days. Regular biochemical monitoring was undertaken with measurement of acid–base status and ammonia levels. In contrast to their older brother, these male siblings had unremarkable neonatal periods with no significant clinical or biochemical concerns, demonstrating that in a neonate known to be affected with CAVA deficiency, early intervention can be instituted to minimise the risk of metabolic decompensation in the neonatal period.

## Key Points

- We describe the clinical course of two sibling infants antenatally diagnosed with carbonic anhydrase VA (CAVA) deficiency. They were monitored and managed in the newborn period without decompensation.
- Neonatal management included regular breastfeeds with supplementary top‐ups to ensure optimal nutrition and administration of carglumic acid (100 mg/kg daily) for 5 days. Regular biochemical monitoring was undertaken with measurement of acid–base status and ammonia levels.
- In neonates known to be affected with CAVA deficiency, early intervention can be instituted to minimise the risk of metabolic decompensation in the neonatal period.

## 1 Introduction

Carbonic anhydrase VA (CAVA) deficiency (OMIM 615751) is an autosomal recessive disease first described in 2014 by van Karnebeek et al., who reported four patients with biallelic pathogenic variants in the *CA5A* gene [1] (OMIM 114761). *CA5A* encodes for the liver mitochondrial carbonic anhydrase Va enzyme that catalyses the reversible conversion of carbon dioxide to bicarbonate (CO_2_ + H_2_0 ⇌ HCO_3_^−^ + H^+^). All symptomatic patients to date have presented with poor feeding, encephalopathy and a mixed acid–base status with hyperammonaemia, hyperlactaemia and ketonaemia/ketonuria [1, 2, 3, 4, 5]. The biochemistry observed reflects deficiency of the mitochondrial enzymes that use bicarbonate as a substrate, most significantly carbamoylphosphate synthetase 1 (CPSI) and pyruvate carboxylase (PC) in addition to propionyl CoA carboxylase (PCC) and 3‐methylcrotonyl carboxylase (3‐MCC). CPSI catalyses the rate‐limiting step in the conversion of waste nitrogen to urea where deficiency in the synthesis of carbamoyl phosphate from ammonium and bicarbonate results in hyperammonaemia. PC catalyses the conversion of pyruvate to oxaloacetate, which is an essential anaplerotic substrate for the tricarboxylic acid cycle. Cases have also demonstrated elevated glutamine and alanine and low‐to‐normal citrulline in plasma, suggestive of a proximal urea cycle disorder [4]. Urine organic acid profiles have revealed elevations in 3‐hydroxypropionate, propionylglycine, methylcitrate, lactate, beta‐hydroxybutyrate and acetoacetate, mimicking multiple carboxylase deficiency. A normal lysine level, elevated glutamine and decreased/normal levels of citrulline suggest the secondary impairment of CPSI is the predominant driver of the biochemical profile [1, 6]. Diagnosis is confirmed with establishment of biallelic pathogenic variants in *CA5A* [1].

Fewer than 20 patients have been reported in the literature, with most cases presenting in the first few days of life. Management of acute decompensation has been targeted to the presenting clinical and biochemical features [1, 2]. Protein restriction, provision of caloric support in the form of glucose‐containing intravenous fluids and lipids, nitrogen scavengers, and in some cases haemofiltration and/or carglumic acid have been the mainstay of treatment. Longer‐term management varies, with some patients maintained on protein‐restricted diets, nitrogen scavengers and/or carglumic acid and others only treated during episodes of catabolism with ‘sick day’ plans that provide additional protein‐free calories, and targeted management of hyperammonaemia [1, 5, 6]. Prognosis appears to be largely reassuring, with approximately 2/3 of patients only experiencing a single episode of decompensation, and most having unremarkable neurodevelopmental outcomes, although recurrent hyperammonaemia [6] and fatalities have been reported [5, 7].

## 2 Pedigree

See (Figure 1).

**FIGURE 1:**
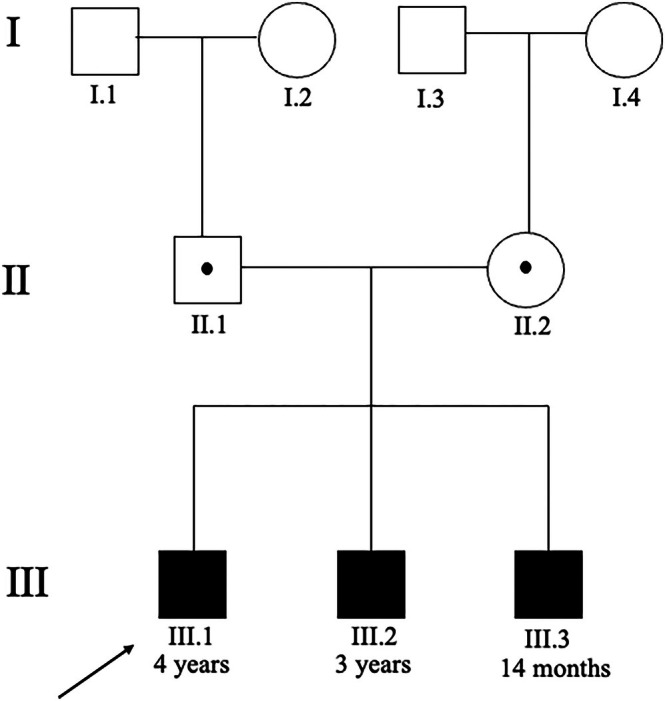
Pedigree.

## 3 Case Series

Male siblings (III.2, III.3) are the second and third child to non‐consanguineous parents of Caucasian maternal North‐American and paternal Russian background (see Figure 1). The proband (III.1) had been born at term after a naturally conceived and uncomplicated pregnancy. He appeared well with reportedly normal breastfeeding until day four of life when he presented with lethargy, feeding difficulties and irritability. Initial investigations revealed a partially compensated high anion‐gap metabolic acidosis and hyperammonaemia (see Table 1).

**TABLE 1:** Investigation results (III.1)—Day 4 of life.

Investigation	Result	Reference
pH	7.18	7.32–7.43
pCO_2_ (mmHg)	18	38–54
HCO_3_ (mmol/L)	6	15–29
Base excess (mmol/L)	−22	−3 – +3
Anion gap (mmol/L)	29	4–13
Lactate (mmol/L)	11.7	0.5–2.2
Ammonia (μmol/L)	450	< 50
Glucose (mmol/L)	4.7	3–6
Alanine transaminase (U/L)	32	< 25
Aspartate transaminase (U/L)	82	< 51

Management included fluid resuscitation, intravenous glucose and lipid provision, nitrogen scavengers, cofactor supplementation, carnitine and sodium bicarbonate infusion. Haemofiltration was required. Stability was achieved over 5 days with discharge on Day 10 of life.

Urine organic acid profile was consistent with severe ketoacidosis and lactic acidosis. Hyperammonaemia with lactic and ketoacidosis suggested a possible organic aciduria [1] or CAVA deficiency [3, 4, 5]. However, hypoglycaemia and the characteristic urine organic acid profile reported previously were not present [1, 2] in our case. This includes elevated organic acids from impaired carboxylase enzyme function with propionylcarnitine, 3‐hydroxypropionate and methylcitrate from propionyl CoA carboxylase dysfunction and 3‐methylcrotonylcarnitine and 3‐hydroxyisovalerate from 3‐methyl crotonyl CoA carboxylase dysfunction [1]. Relevant investigation results are shown in Table 2 including plasma acylcarnitine profile.

**TABLE 2:** Metabolic biochemical results (III.1)—Day 4 of life.

Metabolite	Result	Reference
Urine organic acids		
Lactate (mmol/mol creatinine)	> 2000	< 230
3‐hydroxybutyrate (mmol/mol creatinine)	> 1000	< 28
2‐ketoisovalerate (mmol/mol creatinine)	97	< 2
2‐ketoisocaproate (mmol/mol creatinine)	72	< 2.5
Plasma acylcarnitine profile		
3‐hydroxybutyrylcarnitine (μmol/L)	0.88	< 0.05
Propionylcarnitine (μmol/L)	0.55	< 0.85
Isovalerylcarnitine (μmol/L)	0.12	< 0.85
Other—plasma		
Methylmalonate (μmol/L)	0.08	< 1.50
Glutamine (μmol/L)	1000	530–960
Alanine (μmol/L)	500	230–410
Citrulline (μmol/L)	19	10–30
Lysine (μmol/L)	160	110–270

Molecular testing via a bioinformatic gene panel identified compound heterozygous variants in *CA5A* (see Table 3).

**TABLE 3:** Genetic testing results (III.1).

Gene	Variant	Classification	Inheritance
*CA5A*	c.198_208delinsACCCGG (p.Ile67Profs*13)	Pathogenic	Paternal
*CA5A*	c.454G>A (p.Ala142THr)	Variant of uncertain significance	Maternal

The paternally‐inherited variant creates a premature translational stop signal and was classified as a pathogenic variant per the American College of Medical Genetics and Genomics (ACMG) classification criteria [8]. The maternally‐inherited variant has not been reported in the medical literature to date. As the biochemical profile supports the diagnosis of CAVA deficiency, the maternally carried *CA5A* VUS is suggested to be disease‐causing.

Following natural conception of their second and third children, they elected to undergo prenatal targeted genetic testing via amniocentesis at 16 weeks' gestation through an accredited laboratory for prenatal diagnosis. Both pregnancies confirmed an affected fetus. A postnatal management plan for each infant was compiled. The decision was made to deliver at a tertiary neonatal unit co‐located with a quaternary paediatric hospital, so that rapid escalation of care could be achieved if severe metabolic decompensation developed.

### 3.1 III.2

Labour was induced at 38 weeks gestation for maternal obstetric indications. The male infant was born in good condition and had some mild respiratory distress at birth, treated with a short period of continuous positive airway pressure. His birth weight was 3486 g (44th centile), length 50 cm (33rd centile) and head circumference 34 cm (22nd centile). Clinical examination was unremarkable.

III.2 was admitted to the neonatal nursery but predominantly cared for on the maternity ward to allow maternal bonding. He was commenced on a strict oral feeding regime of breast feeding with supplementary expressed breast milk and standard infant formula in accordance with local protocols for a well, term neonate.

Carglumic acid was administered at a dose of 100 mg/kg/day for 5 days. Regular clinical and biochemical monitoring was undertaken from 12 h of life with measurements of venous blood gases and ammonia levels, initially 12‐hourly then decreasing to 24–48 hourly as he remained well. He was discharged from hospital at 5 days of life, without having evidenced any significant biochemical derangements.

### 3.2 III.3

Delivery was planned at a tertiary obstetric unit with an induction of labour at 38 + 3 weeks gestation. The male infant was born in good condition with APGAR of 8^1^ and 9^5^ requiring no resuscitation. His birth weight was 3368 g (37th centile), length 51 cm (62nd centile) and head circumference 35.5 cm (43rd centile). He had an unremarkable clinical examination.

Similar to his older brother, III.3 was admitted to the neonatal nursery with predominant care on the maternity ward and was managed with a strict oral feeding regime, carglumic acid 100 mg/kg/day for 5 days and regular biochemical monitoring with venous blood gases and ammonia levels. He also remained stable without evidence of biochemical derangement and was discharged at 5 days of life.

## 4 Discussion

Genetic diagnosis in a proband permits carrier testing in the parents to inform risk of recurrence and provides the ability to offer prenatal diagnosis in future pregnancies. This is particularly relevant in a condition such as CAVA deficiency where many patients have essentially unremarkable biochemistry when well, and where markers for biochemical testing of amniotic fluid have not been established [9]. In this case, the proband's siblings were identified to have the same biallelic variants in *CA5A*. Each sibling had a normal extended newborn bloodspot screening result.

The perinatal management of an antenatally diagnosed infant affected with CAVA deficiency has not yet been reported. Proposed recommendations include delivery in hospital with monitoring for at least 3 days of ammonia levels, serum lactate, serum glucose and blood gases [9]. Off‐label use of carglumic acid in acute, undifferentiated hyperammonaemic decompensations has been described, with anecdotal reports of rapid improvement in hyperammonaemia. The presumed mechanism is that of enhancement of CPSI activity despite decreased availability of bicarbonate [1]. As our service does not use carglumic acid regularly, it was obtained for the birth of these infants and the decision was made to administer five once‐daily doses of 100 mg/kg to the patient prophylactically, rather than await potential metabolic decompensation. Practical difficulties in administration of carglumic acid included the palatability of the medication. The manufacturer's recommendation is to dissolve the tablets in water and administer immediately prior to feeding; however, the best tolerance for our patients was obtained when the tablets were dissolved in an equivalent amount of expressed breast milk. Carglumic acid was used for each sibling infant and outcomes may have been due to its use, regular feeding and monitoring, or all of these factors. Practical challenges to regular venous blood samples in a newborn baby were also noted, as invasive monitoring lines were not indicated.

Prognostication and optimal long‐term medical management remain uncertain in CAVA deficiency. While early reports suggested that most children have only one significant decompensation, commonly within the first few days or months of life (presumably reflecting a period of metabolic vulnerability) and then follow an uncomplicated neurodevelopmental trajectory, more recent reports have demonstrated recurrent episodes of hyperammonaemia into middle childhood, despite optimal medical management [1, 2, 3, 4, 6]. Purported mechanisms for the relatively benign trajectory of CAVA deficiency include possible compensation by carbonic anhydrase Vb (CAVB; another mitochondrial carbonic anhydrase) and the CAVA‐independent production of bicarbonate via the same biochemical reaction that is catalysed by CAVA, albeit at a much slower rate [1, 10]. Of note, CAVB deficiency has not been reported to result in human disease and so is not included on most gene panels, but concomitant deficiency could conceivably explain the rapidly progressive fatal course seen in two previously reported individuals despite optimal management [1, 5].

In our family's case, antenatal diagnosis facilitated careful obstetric and neonatal management with avoidance of metabolic decompensation, although with short‐term associated burdensome medical interventions. In the sibling's older brother, the initial presentation was managed as an undiagnosed urea cycle disorder or organic acidaemia with dietary protein restriction and carnitine supplementation until these were excluded. By contrast, these infants have been exclusively breastfed since discharge and are not on any regular medications. The proband (III.1) is now 4 years old and siblings are 3 years (III.2) and 14 months old (III.3). The proband has not experienced any recurrence of hyperammonaemia or apparent neurodevelopmental compromise. Sick day protocols have been provided for use during illness. The siblings have remained well with normal development and no evidence of decompensation. We remain hopeful they will continue to follow the same course.

## 5 Conclusion

Sibling infants known to be at risk of severe metabolic decompensation were pre‐emptively managed in the newborn period. Expectant preparation, monitoring and management avoided metabolic decompensation in the newborn period and suggests an approach to management of infants diagnosed antenatally with CAVA deficiency.

## Author Contributions

S.M., T.M. and K.D. prepared the original manuscript. All authors provided input into the manuscript. D.C. provided oversight. All authors take responsibility for the content of the article.

## Funding

The authors have nothing to report.

## Ethics Statement

Local human research ethics committee protocols were followed, including obtaining informed consent with a caregiver.

## Consent

Informed consent for preparation of this case report was obtained with a caregiver for each case subject.

## Conflicts of Interest

The authors declare no conflicts of interest.

## Data Availability

The data that support the findings of this study are available on request from the corresponding author. The data are not publicly available due to privacy or ethical restrictions.

## References

[1] C. D. van Karnebeek , W. S. Sly , C. J. Ross , et al., “Mitochondrial Carbonic Anhydrase VA Deficiency Resulting From CA5A Alterations Presents With Hyperammonemia in Early Childhood,” American Journal of Human Genetics 94, no. 3 (2014): 453–461. 24530203 10.1016/j.ajhg.2014.01.006 PMC3951944

[2] C. Diez‐Fernandez , V. Rüfenacht , S. Santra , et al., “Defective Hepatic Bicarbonate Production due to Carbonic Anhydrase VA Deficiency Leads to Early‐Onset Life‐Threatening Metabolic Crisis,” Genetics in Medicine 18, no. 10 (2016): 991–1000. 26913920 10.1038/gim.2015.201

[3] A. Olgac , C. S. Kasapkara , M. Kilic , et al., “Carbonic Anhydrase VA Deficiency: A Very Rare Case of Hyperammonemic Encephalopathy,” Journal of Pediatric Endocrinology and Metabolism 33, no. 10 (2020): 1349–1352. 32809955 10.1515/jpem-2020-0117

[4] A. Marwaha , J. Ibrahim , T. Rice , et al., “Two Cases of Carbonic Anhydrase VA Deficiency—An Ultrarare Metabolic Decompensation Syndrome Presenting With Hyperammonemia, Lactic Acidosis, Ketonuria, and Good Clinical Outcome,” JIMD Reports 57, no. 1 (2021): 9–14. 33473334 10.1002/jmd2.12171 PMC7802620

[5] R. Konanki , R. R. D. Akella , N. Panigrahy , D. K. Chirla , S. Mohanlal , and R. Lankala , “Mitochondrial Carbonic Anhydrase VA Deficiency in Three Indian Infants Manifesting Early Metabolic Crisis,” Brain & Development 42, no. 7 (2020): 534–538. 32381389 10.1016/j.braindev.2020.04.007

[6] C. Stockdale , A. Bowron , M. Appleton , R. Richardson , and M. Anderson , “Recurrent Hyperammonaemia in a Patient With Carbonic Anhydrase VA Deficiency,” JIMD Reports 63, no. 6 (2022): 536–539. 36341166 10.1002/jmd2.12322 PMC9626664

[7] F. Baertling , M. Wagner , T. Brunet , et al., “Fatal Metabolic Decompensation in Carbonic Anhydrase VA Deficiency Despite Early Treatment and Control of Hyperammonemia,” Genetics in Medicine 22, no. 3 (2020): 654–655. 31641285 10.1038/s41436-019-0677-9

[8] S. Richards , N. Aziz , S. Bale , et al., “Standards and Guidelines for the Interpretation of Sequence Variants: A Joint Consensus Recommendation of the American College of Medical Genetics and Genomics and the Association for Molecular Pathology,” Genetics in Medicine 17, no. 5 (2015): 405–424. 25741868 10.1038/gim.2015.30 PMC4544753

[9] C. van Karnebeek and J. Häberle , “Carbonic Anhydrase VA Deficiency,” in GeneReviews(), ed. M. P. Adam , J. Feldman , G. M. Mirzaa , R. A. Pagon , S. E. Wallace , and A. Amemiya (University of Washington, 1993–2025). 25834911

[10] B. Singanamalla , A. Saini , S. Attri , R. Suthar , and K. Mukhopadhyay , “Carbonic Anhydrase‐VA Deficiency: A Close Mimicker of Urea Cycle Disorders,” Annals of Indian Academy of Neurology 24, no. 5 (2021): 820–821. 35002167 10.4103/aian.AIAN_563_20 PMC8680920

